# Acute myocardial infarction due to isolated spontaneous coronary artery dissection in the first septal branch: a case report

**DOI:** 10.1093/ehjcr/ytaf339

**Published:** 2025-07-14

**Authors:** Kosuke Tanimura, Ryo Matsutera, Kenji Nakajima, Hideyuki Takaoka

**Affiliations:** Department of Cardiology, Aijinkai Takatsuki General Hospital, 1-3-13 Kosobe-cho, Takatsuki, Osaka 569-1192, Japan; Department of Cardiology, Aijinkai Takatsuki General Hospital, 1-3-13 Kosobe-cho, Takatsuki, Osaka 569-1192, Japan; Department of Cardiology, Aijinkai Takatsuki General Hospital, 1-3-13 Kosobe-cho, Takatsuki, Osaka 569-1192, Japan; Department of Cardiology, Aijinkai Takatsuki General Hospital, 1-3-13 Kosobe-cho, Takatsuki, Osaka 569-1192, Japan

**Keywords:** Acute myocardial infarction, Case report, First septal branch, Intravascular ultrasound, Spontaneous coronary artery dissection

## Abstract

**Background:**

Myocardial infarction due to isolated spontaneous dissection in the septal branch of the coronary arteries is a rare cause of myocardial infarction with non-obstructive coronary artery disease and is challenging to diagnose.

**Case summary:**

A 61-year-old woman presenting with acute chest pain exhibited slight ST-segment elevation in leads V1–3 on electrocardiography. Echocardiography revealed mild hypokinetic septal wall motion. Contrast-enhanced computed tomography demonstrated a low-contrast area in the posterior septum. Emergency coronary angiography revealed delayed flow and stenosis in the first septal branch of the left anterior descending coronary artery. Intravascular ultrasound demonstrated a circumferential haematoma without an intimal tear or double lumen at the stenotic site. No balloon angioplasty or stent implantation was required because the flow delay in the septal branch disappeared after intravascular ultrasound procedure. The patient was conservatively managed with single antiplatelet therapy, calcium channel blocker, and statin. After 1 month, follow-up coronary computed tomography angiogram confirmed vascular patency of the first septal branch without narrowing, and the patient showed no recurrence.

**Discussion:**

Myocardial infarction due to isolated spontaneous coronary dissection in the septal branch of the coronary arteries is an often overlooked cause of myocardial infarction with non-obstructive coronary artery disease. Therefore, intravascular imaging and contrast-enhanced computed tomography should be performed in cases of myocardial infarction with a non-obstructive coronary artery to locate the site of infarction and identify the cause.

Learning points

- Spontaneous coronary artery dissection in the isolated septal branch is an exceptionally rare condition that may be missed as a cause of myocardial infarction with non-obstructed coronary arteries.
- In addition to coronary angiography, contrast-enhanced computed tomography may be helpful for accurately identifying the site of infarction.
- Intravascular imaging can help to identify dissections and haematomas in small or atypical branches, such as the septal branches.

## Introduction

Myocardial infarction associated with only the first septal branch of the left anterior descending coronary artery is rare. The mechanisms of isolated septal myocardial infarction are coronary spasm, embolization, or artery dissection.^1–3^ Spontaneous coronary artery dissection (SCAD) is a recognized cause of acute coronary syndrome that disproportionately affects women and has few traditional cardiovascular risk factors.^4,5^ It accounts for a subset of myocardial infarctions with non-obstructive coronary artery (MINOCA) cases, in whom coronary angiography may not reveal significant stenosis. SCAD affecting only the first septal branch is extremely rare and poses diagnostic challenges because initial angiography may yield unclear results.^6^

We report a case of myocardial infarction due to isolated SCAD of the first septal branch, focusing on intravascular ultrasound findings and changes in coronary computed tomography angiography (CCTA).

## Summary figure

**Figure:**
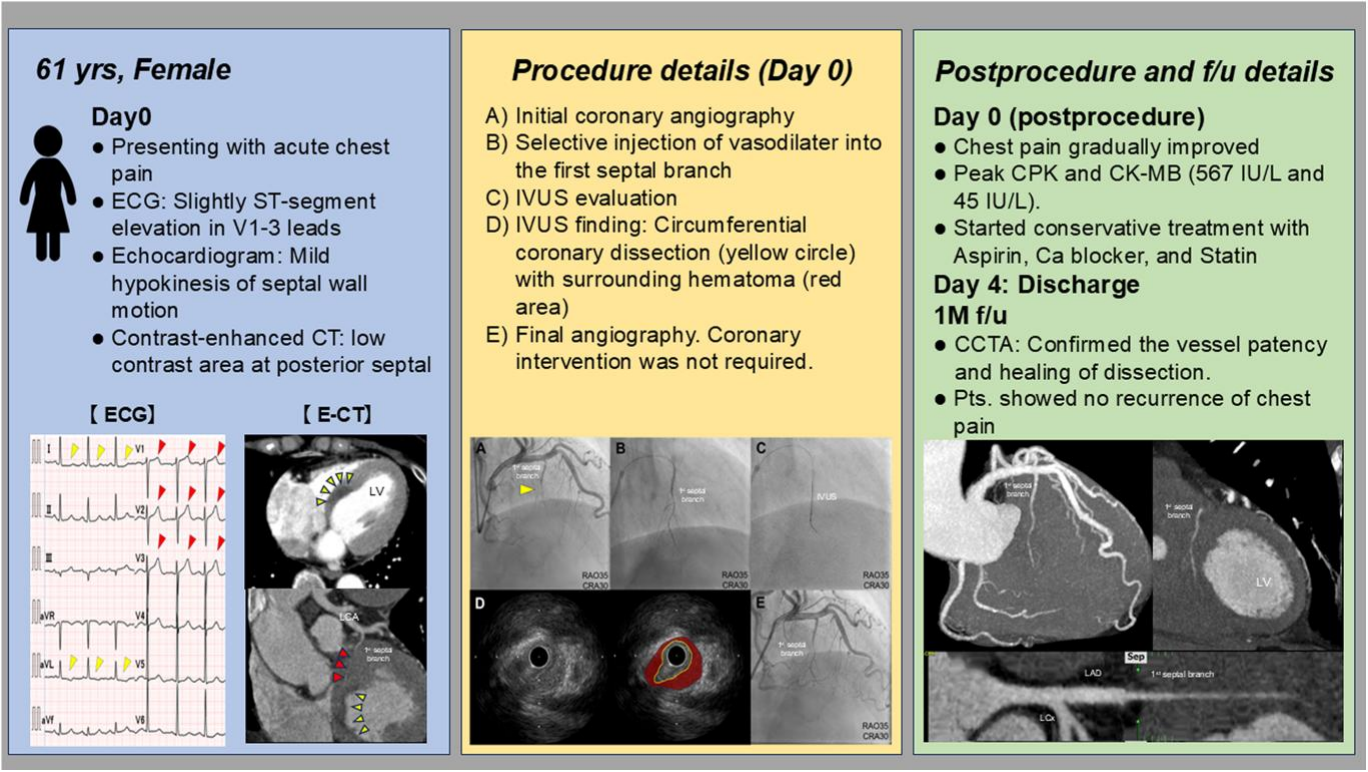


## Case presentation

A 61-year-old woman developed acute chest pain and shortness of breath while on her way home and was admitted to our emergency room because her chest pain worsened after defaecation. The patient had untreated hypertension, a smoking history, and a family history of cardiovascular disease, but no history of diabetes mellitus or dyslipidaemia.

**Figure 1:**
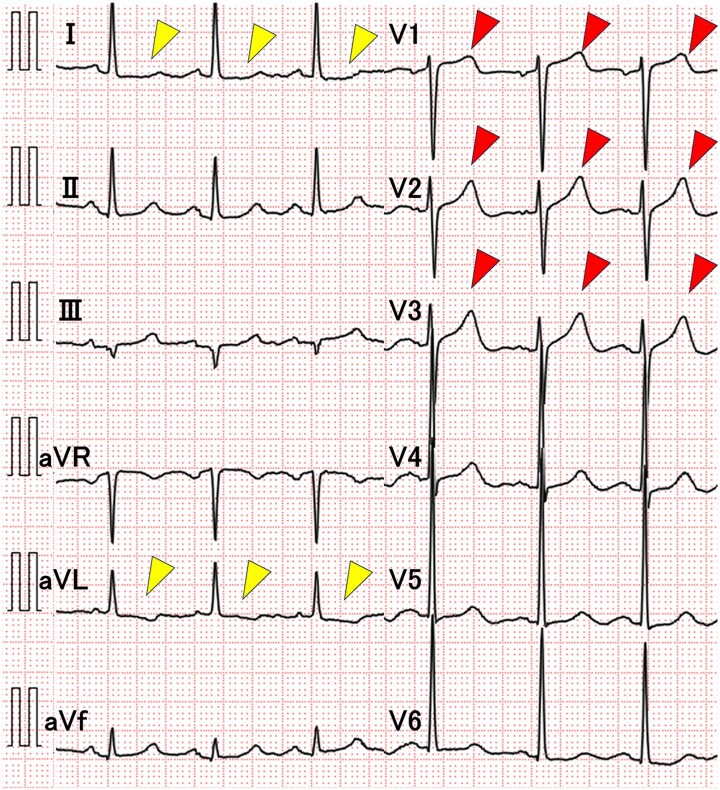
Findings on electrocardiogram. T-wave peaking is observed in leads V1–3 (red arrowheads) and slight ST depression is seen in leads I and aVL (yellow arrowheads).

On examination, her blood pressure was elevated (199/135 mmHg), heart rate was 79 beats per minute, body temperature was 36.4°C, and oxygen saturation was 96% on room air. Physical examination revealed regular heart sounds, without gallops or murmurs. Laboratory tests revealed normal troponin T levels (0.012 ng/mL; normal <0.014 ng/mL) and no elevation in serum creatine kinase (100 IU/L; normal 45–163 U/L) and creatine kinase-MB (15 U/L; normal 0–24 U/L). A 12-lead electrocardiogram demonstrated T-waves peaking in leads V1–3 and a slight ST depression in leads I and aVL (*Figure 1*). Chest radiography revealed no cardiomegaly or pulmonary congestion. Transthoracic echocardiography demonstrated slightly reduced wall motion at the base of the posterior septum, with a preserved ejection fraction of 55%, and no valvular heart disease or pulmonary hypertension.

**Figure 2:**
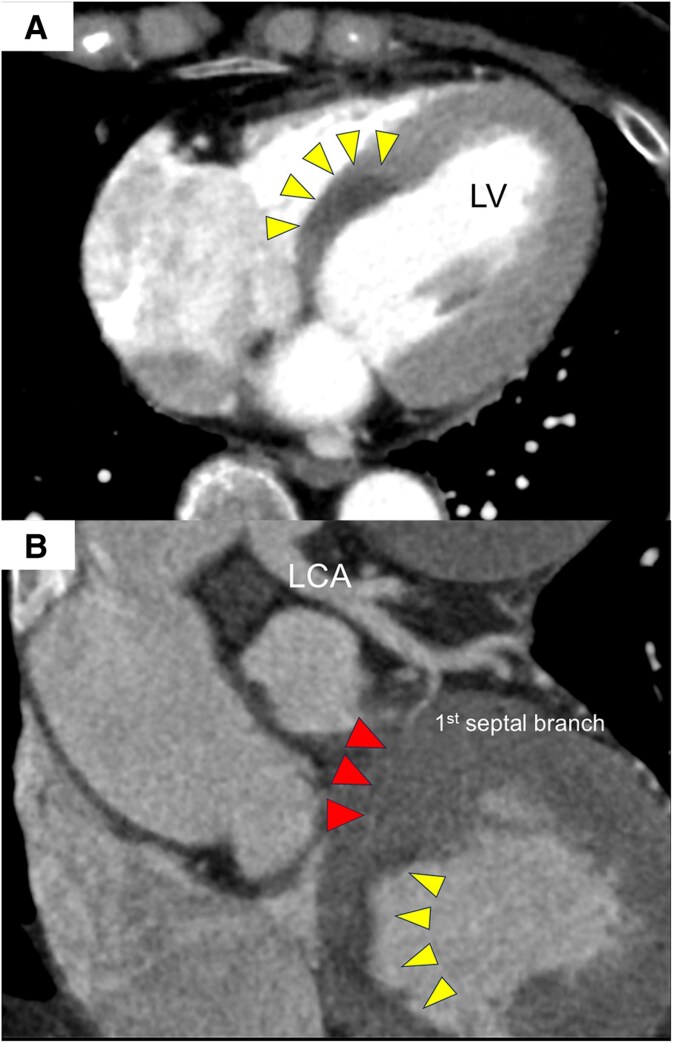
Contrast-enhanced computed tomography findings. (*A*) A contrast-impaired area is seen at the base of the ventricular septum in the arterial phase (yellow arrowheads). (*B*) Stenosis of the perfused septal branch is observed in the low-contrast area (red arrowhead). LCA, left coronary artery; LV, left ventricular.

**Figure 3:**
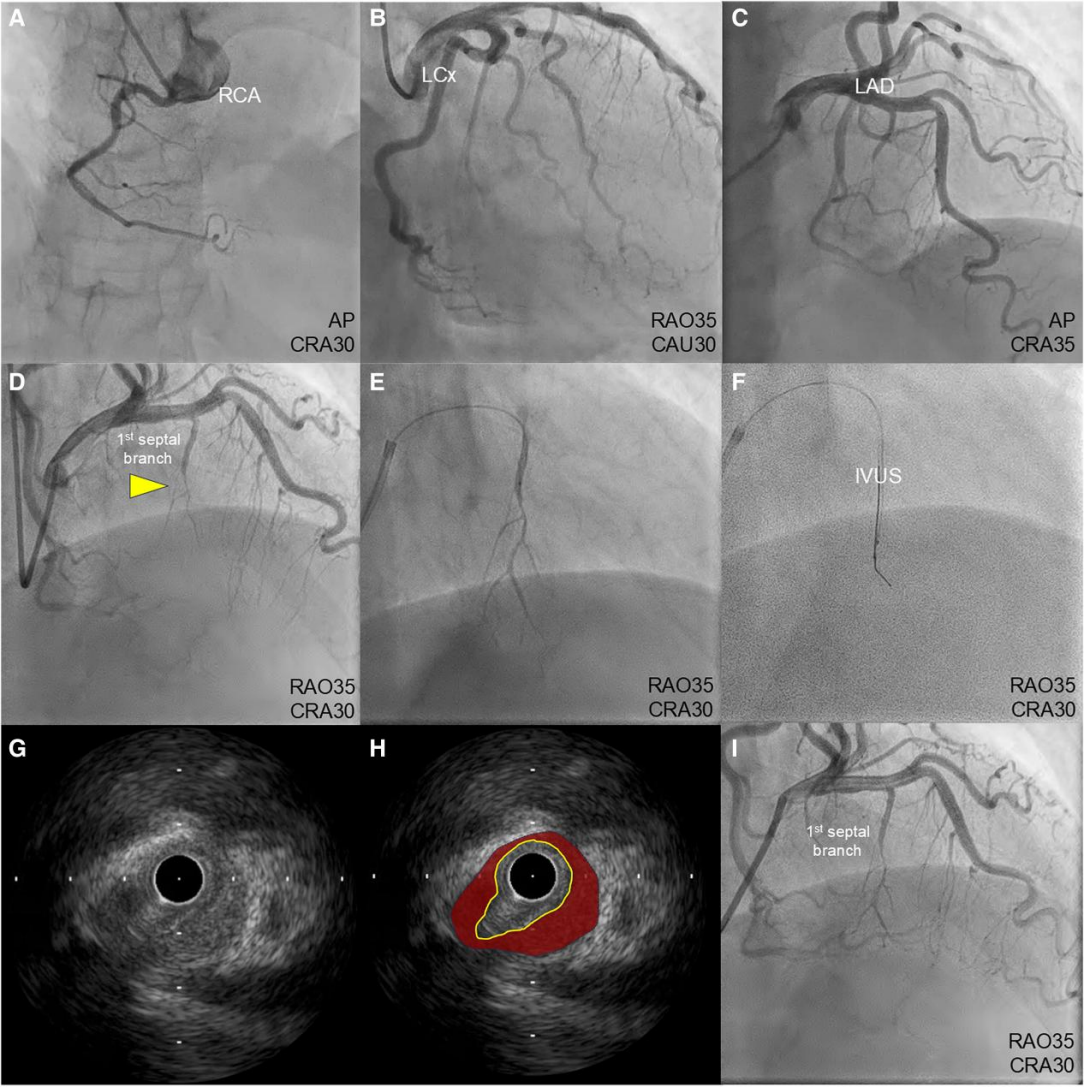
Emergency coronary angiography and intravascular ultrasound findings. Coronary angiography shows (*A–D*) delayed flow and severe stenosis in the first septal branch of left anterior descending coronary artery (yellow arrowhead), without stenosis of any other segment. (*E*) Selective injection of vasodilator into the first septal branch via a microcatheter. (*F*) Manual pullback of the intravascular ultrasound probe. (*G*, *H*) Intravascular ultrasound images of the circumferential dissection of the coronary branch (yellow circle), with a surrounding haematoma (red area). (*I*) Final angiography. IVUS, intravascular ultrasound; LAD, left anterior descending; LCx, left circumflex.

Although acute coronary syndrome was suspected based on electrocardiography (ECG) and echocardiography, electrocardiogram-synchronized contrast-enhanced computed tomography (CT) was performed to exclude acute aortic dissection. While no clear evidence of aortic dissection was found, a contrast-impaired area was observed at the base of the ventricular septum in the arterial phase, along with septal branch stenosis (*Figure 2*). Although the CT suggested a perfusion defect in the posterior septum, the cause of ischaemia could not be definitively identified. Consequently, emergency coronary angiography and intracoronary imaging were performed to investigate the cause of ischaemia. Emergency coronary angiography revealed delayed flow and severe stenosis in the first septal branch of the left anterior descending coronary artery, without stenosis of any other segment (*Figure 3A–D*; Supplementary material online, *Video S1*). As we suspected vasospasm, isosorbide (1 mg) and nicorandil (2 mg) were selectively injected into the first septal branch via a microcatheter, but the stenosis persisted, despite improved blood flow (*Figure 3E*; Supplementary material online, *Video S2*). Intravascular ultrasound (IVUS) (Navifocus WR®, Terumo, Tokyo, Japan) was performed to evaluate the vascular characteristics (*Figure 3F*). Manual pullback of the IVUS probe revealed a circumferential haematoma without an intimal tear or double lumen at the stenotic site in the septal branch, consistent with a spontaneous intramural haematoma-type SCAD. No dissection was observed in the main trunk of the coronary artery (*Figure 3G, H*; Supplementary material online, *Video S3*). This finding was evident prior to vasodilator selective injection, suggesting that the dissection was due to SCAD rather than an iatrogenic injury.

After the IVUS procedure, the septal branch flow delay disappeared, partly due to the Bougie effect of the IVUS probe passage; thus, balloon angioplasty or stent implantation was not necessary (*Figure 3I*). The postoperative peak serum concentrations of creatine kinase and creatine kinase-MB increased to 567 and 45 IU/L, respectively.

**Figure 4:**
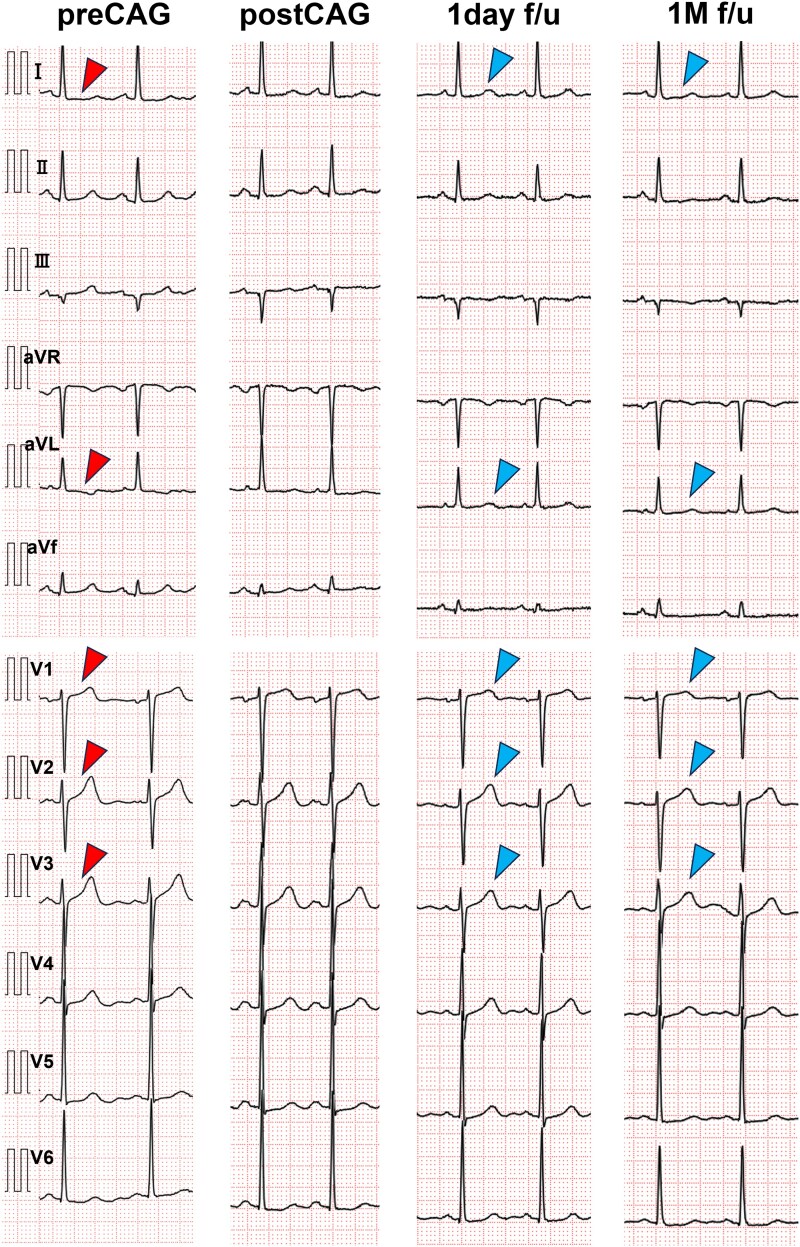
Changes in electrocardiogram findings after the procedure. T-wave peaking in leads V1–3 and slight ST depression in leads I and aVL (red arrowheads) resolved following the procedure (blue arrowheads).

**Figure 5:**
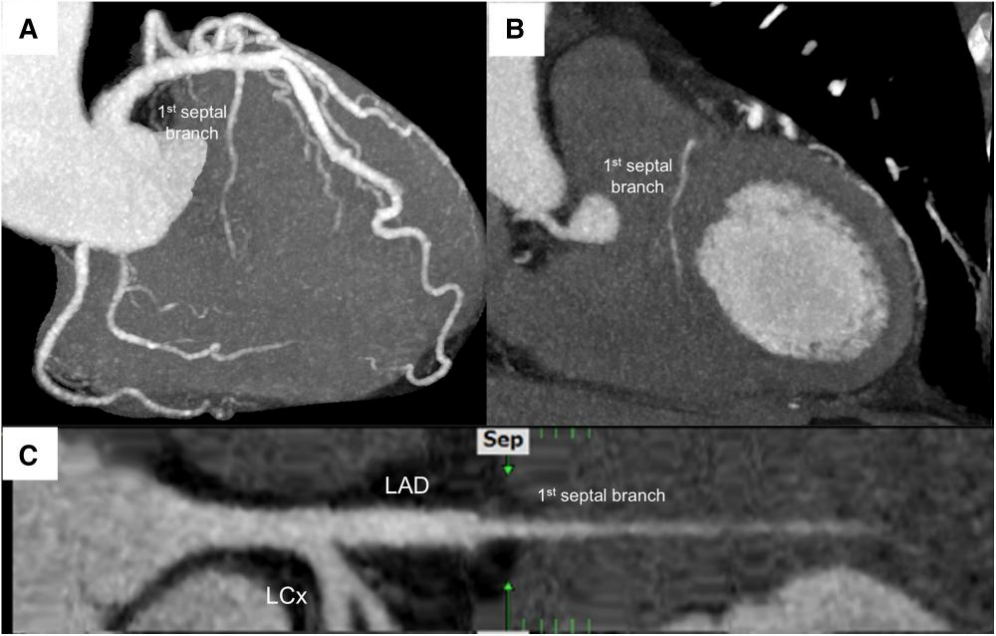
One-month follow-up coronary computed tomography angiogram. (*A*) The first septal branch shows no residual signs of dissection. (*B*) Perfused first septal branch in the infarction site. (*C*) Stretched curved planar reconstruction of the first septal branch.

Based on coronary angiography and IVUS findings, acute myocardial infarction due to isolated SCAD in the first septal branch was diagnosed. Chest pain gradually improved post-examination, and the ECG changes showed improvement the next day (*Figure 4*). The patient was discharged without complications on the fourth day, after receiving antiplatelet (aspirin 100 mg) and lipid-lowering therapy (atorvastatin 10 mg), a calcium channel blocker (nifedipine CR 20 mg), and nicorandil 15 mg. One month later, CCTA confirmed the patency of the first septal branch without narrowing (*Figure 5*).

## Discussion

Myocardial infarction caused by isolated SCAD in the first septal branch of the left descending coronary artery, as occurred in the presented case, is exceptionally rare. Herein, we discuss the implications of this condition and the importance of advanced imaging techniques in its diagnosis.

In general, myocardial infarction related to only the septal branch is rare because this vessel is smaller than the main trunk, and atherosclerotic changes in this branch are considered scarce. In our case, prior to coronary angiography, contrast-enhanced CT with synchronous ECG confirmed the presence of an infarcted lesion, and coronary angiography could then reliably detect septal branch occlusion. However, contrast-CT alone does not provide a definitive assessment of myocardial necrosis. Previous case reports of isolated first septal branch infarcts have emphasized the difficulty of diagnosing the condition using coronary angiography alone and have highlighted the need to demonstrate myocardial damage by using other imaging modalities (e.g. cardiac magnetic resonance imaging [CMRI] or nuclear imaging).^3,6,7^ CMRI, particularly with late gadolinium enhancement, is known to more reliably confirm infarction. Although CMRI with late gadolinium enhancement was not performed in this case due to the patient's stable clinical course and the diagnosis being supported by multimodal evidence, including echocardiography, coronary angiography, intravascular ultrasound findings, and contrast-enhanced CT, performing CMRI would have provided definitive confirmation of the infarcted site.

The current European Society of Cardiology statement emphasizes the importance of considering SCAD in young or middle-aged women with acute coronary syndrome, particularly in the absence of traditional cardiovascular risk factors. Known predisposing factors include hormonal influences (e.g. peripartum period), connective tissue disorders, fibromuscular dysplasia, and systemic inflammatory diseases. Emotional or physical stress is also commonly reported as a trigger.^4,5^ In the present case, although the patient did not exhibit typical features such as connective tissue disease or peripartum status, several contributing factors were identified (including untreated hypertension, a history of smoking, and a family history of cardiovascular disease). Therefore, systemic imaging such as whole-body CT or MRI was not performed. Additionally, the onset of symptoms coincided with physical exertion (defaecation), which may have acted as a mechanical trigger for the dissection. These factors are consistent with known SCAD risk profiles and likely contributed to the pathogenesis in this case. Although the importance of SCAD as a cause of MINOCA is increasingly recognized, it is often not diagnosed because the angiographic findings are ambiguous.^8^ Furthermore, the difficulties in diagnosing SCAD, particularly in small vessels, such as the septal branch, are often missed by standard coronary angiography. In the present case, MINOCA due to vasospasm was suspected because of the absence of significant stenosis on coronary angiography. Therefore, direct injection of a vasodilator was performed, but angiographic findings did not improve. This case highlights the importance of maintaining a high index of suspicion of SCAD, particularly in cases in which coronary angiography does not reveal a clear aetiology. Evaluation with IVUS in our case clarified arterial dissection with haematoma in the first septal branch, which was not obvious on angiography alone. Notably, the IVUS findings were consistent with a spontaneous intramural haematoma-type SCAD, as no intimal flap or double lumen was observed. This subtype of SCAD is becoming increasingly recognized but may be underdiagnosed without intravascular imaging. Although performing IVUS in small vessels, such as the septal branches, carries potential procedural risks, including retrograde dissection into the main coronary artery,^9^ we carefully advanced the wire and performed the IVUS using a manual pullback technique with minimal manipulation. No procedural complications occurred, and the flow delay resolved following the examination. Previous reports have also highlighted the usefulness of IVUS in small vessels to aid in the diagnosis of SCAD,^10^ suggesting that this approach can be safe and diagnostically useful when performed with caution. Regarding intravascular imaging other than IVUS, a previous report showed that intravascular optical coherence tomography is useful in demonstrating SCAD.^2^ Thus, intravascular imaging is recognized as a valuable tool for distinguishing SCAD from other causes, such as vasospasm and emboli.

Furthermore, although isolated SCAD of the first septal branch is rare, and its long-term prognosis remains uncertain, the recognition of isolated SCAD in septal branches is clinically significant, and accurate follow-up imaging requires adjunctive techniques, other than angiography.^6^ In the present case, CCTA was performed 1 month after the event as a non-invasive follow-up imaging test, which confirmed patency of the septal branch. However, it is important to note that CCTA has limited spatial resolution when identifying subtle features of coronary artery dissection, particularly in small vessels such as the first septal branch. In this case, the initial CCTA did not reveal a clear dissection or intimal flap but only demonstrated localized narrowing of the lumen. Therefore, CCTA may underestimate the presence or extent of dissection, emphasizing that intravascular imaging remains essential for a definitive diagnosis.

Moreover, current treatment strategies for SCAD emphasize spontaneous healing and conservative management. Conservative therapy is generally recommended in hemodynamically stable patients with SCAD, as most lesions heal spontaneously. This approach typically includes antiplatelet therapy (usually aspirin), β-blockers to reduce arterial shear stress, and calcium channel blockers or nitrates for vasospasm or hypertension. Statins may be prescribed if the patient has additional cardiovascular risk factors. Close monitoring is essential to detect potential recurrence or propagation of the dissection during hospitalization. Conversely, revascularization may be considered in cases of ongoing ischaemia, hemodynamic instability, or left main artery involvement. However, percutaneous coronary intervention for SCAD can be technically challenging due to risks such as guidewire entry into the false lumen, dissection propagation, and poor stent apposition in fragile vessels. Coronary artery bypass grafting is rarely indicated but may be considered in cases involving the left main coronary artery or extensive multivessel dissection.^4,5,9^ Regarding antiplatelet therapy for SCAD, the optimal regimen and duration remain controversial due to the lack of high-quality evidence and randomized controlled trials. Previous guidelines suggest that for patients who do not undergo stent implantation, single antiplatelet therapy with aspirin may be sufficient, particularly in cases of mild myocardial infarction. The duration of treatment is traditionally recommended continued for 6 to 12 months.^4^ In the present case, no intervention was necessary as blood flow improved following IVUS, and the patient remained hemodynamically stable throughout hospitalization. As a result, conservative therapy with aspirin monotherapy (due to the patient’s atherosclerotic risks), nifedipine, nicorandil, and statins was successfully employed.

This case highlights the diagnostic and management challenges associated with isolated SCAD of the first septal branch. The rarity of this condition, coupled with the limitations of conventional coronary angiography, underscores the importance of using advanced imaging techniques, such as intracoronary imaging and CCTA. Considering the potential underdiagnosis of SCAD, clinicians should maintain a high degree of suspicion of SCAD in MINOCA cases with unclear angiographic findings.

## Conclusion

Isolated SCAD in the first septal branch is often overlooked in MINOCA. Clinicians should consider adopting intravascular imaging and contrast-enhanced CT in such patients to resolve this diagnostic challenge.

## Supplementary Material

ytaf339_Supplementary_Data

## Data Availability

The data underlying this article are available in the article and its online Supplementary material.

## Lead author biography

Dr. Kosuke Tanimura received his licence to practice medicine at Tokushima University in 2012 and completed a Ph.D. at Kobe University Graduate School of Medicine in 2021. His main areas of research are intravascular coronary imaging and interventional cardiology, including coronary artery and structural heart diseases.
